# Soft Radio-Frequency Identification Sensors: Wireless Long-Range Strain Sensors Using Radio-Frequency Identification

**DOI:** 10.1089/soro.2018.0026

**Published:** 2019-02-11

**Authors:** Lijun Teng, Kewen Pan, Markus P. Nemitz, Rui Song, Zhirun Hu, Adam A. Stokes

**Affiliations:** ^1^ The School of Engineering, Institute for Integrated Micro and Nano Systems, The University of Edinburgh, The King's Buildings, Edinburgh, United Kingdom.; ^2^ Microwave and Communication Systems Group, School of Electrical and Electronic Engineering, University of Manchester, Manchester, United Kingdom.; ^3^ Department of Computer Science and Engineering, University of Michigan, Ann Arbor, Michigan.

**Keywords:** soft sensing, RFID, antenna, wireless, passive

## Abstract

Increasing amounts of attention are being paid to the study of *Soft Sensors* and Soft Systems. *Soft Robotic Systems* require input from advances in the field of *Soft Sensors*. Soft sensors can help a soft robot to perceive and to act upon its immediate environment. The concept of integrating sensing capabilities into soft robotic systems is becoming increasingly important. One challenge is that most of the existing soft sensors have a requirement to be hardwired to power supplies or external data processing equipment. This requirement hinders the ability of a system designer to integrate soft sensors into soft robotic systems. In this article, we design, fabricate, and characterize a new soft sensor, which benefits from a combination of radio-frequency identification (RFID) tag design and microfluidic sensor fabrication technologies. We designed this sensor using the working principle of an RFID transporter antenna, but one whose resonant frequency changes in response to an applied strain. This new microfluidic sensor is *intrinsically stretchable* and can be reversibly strained. This sensor is a *passive* and *wireless* device, and as such, it does not require a power supply and is capable of transporting data without a wired connection. This strain sensor is best understood as an RFID tag antenna; it shows a resonant frequency change from approximately 860 to 800 MHz upon an applied strain change from 0% to 50%. Within the operating frequency, the sensor shows a standoff reading range of >7.5 m (at the resonant frequency). We characterize, experimentally, the electrical performance and the reliability of the fabrication process. We demonstrate a pneumatic soft robot that has four microfluidic sensors embedded in four of its legs, and we describe the implementation circuit to show that we can obtain movement information from the soft robot using our wireless soft sensors.

## Introduction

### Soft sensors

*Soft Sensors* is an application-driven research field and has received much attention in recent decades. A soft sensor is a measuring tool that is made of a single polymer or a mixture of soft polymers. These soft polymers are usually *softer* than the materials they interact with, which make them inherently safe. One prominent example is electronic skin. Electronic skin is also called “second skin” referring to its softness; electronic skin is *softer* than human skin and makes it to an ideal substructure for sensor arrays.^1^ A close relative of the field *Soft Sensors* is the field of *Soft Robotics*. One motivating factor for using *Soft Robotics* is the fact that the materials of the robots are *softer* than the environments with which they interact, and this compliance makes them ideal for applications involving human–robot interaction.^2–4^

Autonomous agents that *perceive* and act upon their immediate environment (robots) are influenced by sensor research. Sensor research impacts a robots' sensor modalities, sensor accuracy, or measurement range besides others. The concept of integrating sensing capabilities at low cost has also become increasingly important, especially for certain subgroups in robotics, namely soft robotics, modular robotics, and swarm robotics. In such cases, lower cost systems are motivated by *design for manufacturability* allowing one to commercialize or to scale a robot system. In the past decade, the community has started to make soft robotic actuators, such as (1) ionic actuators,^5–7^ (2) pneumatic actuators,^8–11^ or (3) dielectric actuators^12–14^ to just name a few. These actuators can benefit from soft sensors from feedback force control.

Kim *et al.*^15^ have proposed a stretchable sensor that is made of curvilinear silicon ribbons in an elastic substrate. This novel work allows high-precision complex active electronics to be densely integrated over a few square centimeters area while achieving high degrees of stretchability, bendability, and twistability. Park *et al.*^16^ designed a soft artificial skin with multiple layers of microscale liquid metal channels embedded in an elastomeric substrate to detect multiaxial strain and normal pressure. Single-core copper wire was used in the above researches to interconnect an external data-logger. Other research work, such as Tabatabai *et al.*,^17^ Park *et al.*,^18^ and Majidi *et al.*^19^ have also used similar connection methods.

Although the above-mentioned soft sensors show a promising future for stretchable electronics, they are active sensors that require a power supply and signal processing capabilities, such as a microcontroller with analog–digital converters. As reported in the study of Teng *et al.*,^20^ each of these sensing systems has drawbacks in the interconnection to other devices, and these limitations hinder the uptake of the soft sensor devices into integrated systems. Embedding several such sensors in an untethered soft robotic system is difficult due to power consumption and practical considerations in wiring.

Stretchable antennas that have great potential to be used as wireless soft sensors have been reported in recent years. Stretchable liquid metal antennas have been presented by Cheng *et al.*,^21,22^ Kubo *et al.*,^23^ and So *et al.*^24^ Although these antennas have shown fascinating electronic and mechanic properties, they require coaxial radio frequency (RF) connector or hardwiring to connect to external data processing equipment, which limits their capability of being implemented in sensing applications such as movement monitoring.

Huang *et al.*^25^ mentioned the idea of integrating a measurement chip in stretchable antennas, but the researchers did not provide the solution of integration. Kim *et al.*^26^ demonstrated epidermal electronics using near-field communication; this wireless sensor is biocompatible and achieves sensor reading ranges of a few centimeters. Cheng and Wu^27^ also proposed a reversibly stretchable, large-area wireless strain sensor, which can work wirelessly >5 m with a strain limitation of 15%. These studies pave the way for making wireless stretchable antennas that can be used as soft sensors in soft robotic systems.

### Design of a new soft radio-frequency identification sensor

In this article, we present the design, fabrication, and characterization of a long-range monitoring wireless strain sensor, which is capable of remotely detecting high tensile dynamic strain up to 50% at >7.5 m. This sensor benefits from a combination of radio-frequency identification (RFID) technology and microfluidic electronics fabrication technology. The proposed sensor is also a configurable RFID tag antenna. The RFID integrated circuit chip we integrated in the proposed sensor has very small dimensions (0.4 mm × 0.4 mm × 0.2 mm), so it will not affect the deformability of the sensor.

The electronic properties of this antenna are highly sensitive to mechanical strains at the frequency of operation (800–860 MHz). This sensor can withstand repeated mechanical strain while retaining electrical functionality and can return to its original state after stretching. In comparison to existing stretchable sensors, our sensor is a completely passive and wireless device. This RFID sensor can be embedded into soft robots without requiring any power consumption from the original robotic device. The feature of wireless power extends the lifetime of the device, removing the need for changing batteries, or integrating an energy harvesting system.

Additionally, most of existing gallium-based liquid alloy sensors were designed based on measuring the resistance change of the liquid metal, but the long-time stability of these sensors will be affected by the oxidation of gallium (gallium-based liquid alloy is easily oxidized). Our proposed RFID strain sensor was designed based on resonant frequency change upon strain. Benefiting from the high electrical conductivity of Galinstan, the skin depth (a measure of how closely electric current flows along the surface of a material) of Galinstan under 800–860 MHz is only a few micrometers. Even though the Galinstan in the channels has a thin oxide layer at the surface, the oxide will not have a large effect on the resonant frequency of the sensor.

In this work, we describe the potential of integrating the proposed RFID sensor into soft robots, which have pneumatic actuators. Most of the existing soft sensors have a requirement to be hardwired to power supplies or to signal processing equipment. This dependence on electrical tethers limits the practical use of soft sensors in many promising applications in real-world conditions. Although, recently, researchers have presented some fully untethered soft robotic systems,^28–31^ most of them require bulky onboard components, such as batteries, microprocessors, pumps, or motors. These onboard components add additional weight on the soft systems, and thereby, the systems need to carry more powerful actuators.

In this work, we intended to develop untethered soft sensors, which can work wirelessly by dispensing, entirely, with the need for onboard power and processors. The proposed sensor can be fully embedded into soft robots without adding any other devices or additional electrical tethers, so that one can eliminate the wire and weight pressures on soft robotic systems. The relatively long measurement working range can allow the soft robots to move freely in a reasonably large area.

## Principles and Implementation

### RFID technology

RFID is a low-cost technology that allows the passive wireless communication of data. In recent decades, RFIDs have been used in many projects such as in robot motion sensors,^32^ indoor localization,^33^ and distributed sensor networks.^34^ An RFID system usually consists of a transponder (sometimes called RFID tag) and the reader. The reader transmits electromagnetic waves to reach out to the RFID tag; if the RFID tag is within the operating range, it utilizes the energy of the incoming electromagnetic wave to transmit a new electromagnetic wave that contains RFID tag-specific information. The information usually contains a unique serial number. The reader receives and visualizes the information. RFIDs can be operated in different frequency bands. While the low-frequency (LF) (30–300 KHz) and high-frequency (HF) (3–30 MHz) bands use magnetic flux coupling, the ultra-high-frequency (UHF) (300 MHz–3 GHz) band uses electromagnetic wave coupling. The communication distance of the UHF band is >16 m, which outperforms the LF and HF bands. In this work, the proposed antenna operates in the UHF band.

### Sensor constitutes

The sensor we present in this work consists of an RFID chip and a half-dipole antenna. The antenna consists of eutectic liquid alloy-filled microfluidic channels in an elastic silicone substrate. The eutectic alloy we used is Galinstan. Galinstan^35^ is a family of room temperature liquid eutectic alloys consisting of gallium (Ga), indium (In), and tin (Sn, Stannum in Latin). Gallium-based room temperature liquid alloy has previously been used in strain,^16,36–38^ pressure,^16,36,39^ curvature,^19,40^ and shear^41^ sensors. It can be easily injected into channels of soft structures. The resistance of a channel can be approximated by $R = \frac { \rho }  { A } *l$ ($\rho { \rm{ \;}}$ is resistivity of the liquid metal and *A* and *l* are cross-section area and length of the channel, respectively); so the channel resistance changes through deformation.

The Galinstan we made consists of 68.5% Ga, 21.5% In, and 10% Sn; its melting temperature is −19°C; and its electrical conductivity is 3.46 × 10^6^ S × m^−1^.^36^ Galinstan indicates favorable properties as channel conductor. We observed reliable liquid distributions in the antenna channels despite extreme twisting, stretching, and folding.

We used Ecoflex as the elastic silicone substrate. This silicone material has a Young's modulus of ∼69 kPa.^42^ We chose Ecoflex to be the substrate due to its favorable properties; it is easy to synthesize, easy to handle, low cost, and biocompatible.^43^ In tensile tests with the ASTM D412 system, the maximum elongation of Ecoflex 00-50 (type 00-50) is 980% (defined as the percentage change in length or $\left( { \left( {l - {l_0}} \right) / {l_0}} \right) \times 100 \%$).

### Working principles

**FIG. 1.**
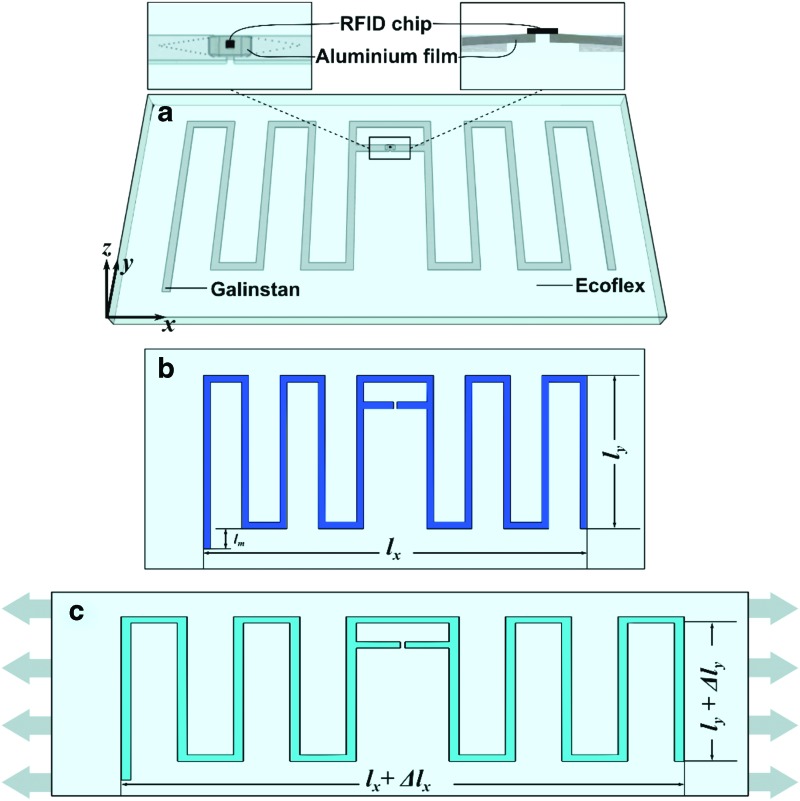
**(a)** Schematic diagram of the stretchable microfluidic sensor. Galinstan is enclosed in Ecoflex substrate while an RFID chip is bonded on a piece of aluminum foil, which is inserted in the channels. Detailed and cross-section figures of the part near RFID chip are presented. Directions of *x*, *y*, and *z* axes are indicated. **(b)** Initial state of the antenna when it is not stretched. The effective antenna length on the *x*-direction and *y*-direction is defined as *l_x_* and *l_y_*, respectively. Notice: the electrical length of the entire antenna is *l_x_* + 10 *l_y_* + *l_m_*. The nonsymmetric part, *l_m_*, is used for fine impedance matching between the antenna and the RFID chip. **(c)** When the soft antenna is stretched, the effective antenna length on the *x*-direction and *y*-direction is defined as *l_x_* + Δ*l_x_* and *l_y_* + Δ*l_y_*, respectively. RFID, radio-frequency identification.

Figure 1a depicts a stretchable meandered half-wave dipole antenna. We embedded an RFID chip into the antenna design. Supplementary Figure S1 indicates specific antenna dimensions.

The RFID chip is bonded on aluminum pads and inserted in between two Galinstan channels. The RFID chip consumes power from received signals and backscatters them without requiring an external power supply depicting a wireless *passive* soft sensor.

The results shown in Figure 1b and c indicate how deformation of an antenna can change its dimensions. If the channel dimensions change, the channel resistances change and therefore the entire antenna and its parameters change. The RFID chip backscatters signals with varying amplitude and phase based on the degree of stretching.

### Implementation in soft robotics

**FIG. 2.**
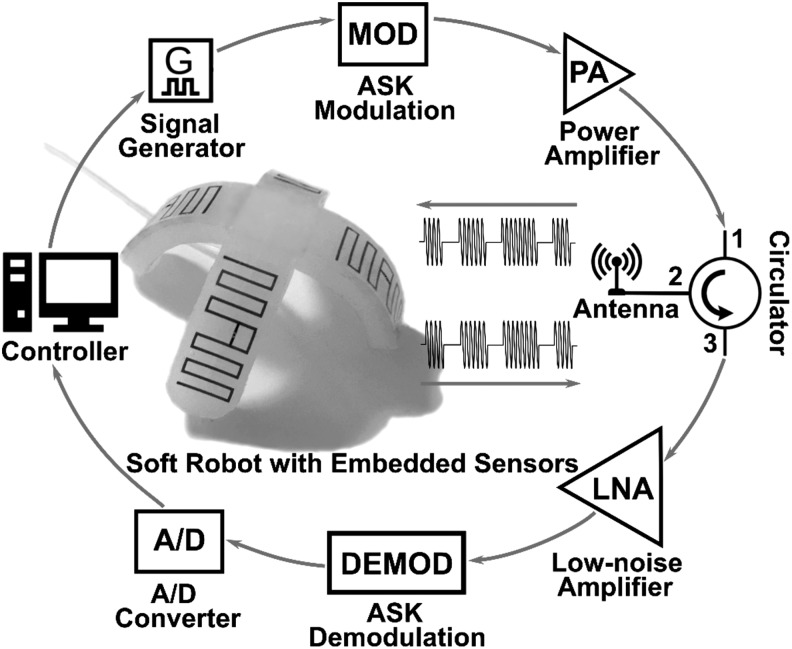
The electronic system for measuring data from a pneumatic soft robot that contains four embedded microfluidic strain sensors.

Figure 2 shows the schematic diagram of our soft RFID sensor implementation. The implementation comprises a pneumatic soft robot with four embedded soft RFID tags, one tag on each robot leg, and the electronic circuitry to transceiver data. The *Controller* uses the *Signal Generator* to generate a HF carrier signal. An *Amplitude Shift Keying* (ASK) module modulates the amplitude of the carrier signal to decode information. A binary “one” consists of a fixed amplitude and frequency; a binary “zero” is a zero-voltage output.

The modulated signal is passed through a power amplifier preparing the signal for transmission. The signal lies on port 1 of a circulator circuit. Port 2 of the circulator outputs the signal to an antenna, therefore broadcasting the signal. The soft RFID tags receive the signal, utilize its energy, and transmit a new signal that incorporates their unique identification information. The antenna receives the signals from the soft RFID tags and outputs them to port 3 of the circulator. The circulator ensures that outgoing and incoming signals do not interfere with one another. The incoming signals from the soft RFID tags pass a low-noise filter, an ASK demodulator, and are ultimately inserted into an analog-to-digital converter. The controller analyses the various signals (distinguishable through their identification information) with regard to their amplitude and phase.

### Antenna design

#### Selection and design of the antenna

The antenna design of our soft RFID sensor is different from other antenna designs because of power and size requirements. The antenna powers the RFID chip with incoming signals; we do not require a power supply. We decided to design the soft RFID sensor as small as possible to attach the RFID tags to even small objects making our sensor more applicable. Since the soft RFID sensors can be attached in any arbitrary orientation, we ensured 360° or *omnidirectional* readouts by using a half-wave dipole antenna design. The effective electrical length of a half-wave dipole antenna is half the wavelength of the operating frequency of the system. This antenna design is commonly used in RF systems with omnidirectional radiation patterns perpendicular to the antenna axis.

In previous work, small antenna sizes were accomplished by using microstrips.^44^ However, they suffered directional readout constraints and low efficiency. Our design uses a meander line antenna (MDA) to achieve long reading ranges and small antenna size. MDA is a derivative of the dipole antenna and achieves high efficiencies^45^ for small antenna sizes.^46,47^

Stretching the soft half-wave dipole antenna as illustrated in Figure 1b causes a change in the dimensions if the antenna is in both the *x* and *y* directions. An increase in strain in the *x*-direction decreases the thickness of the soft polymer, due to the Poisson effect, and also decreases its height by Δ*l_y_*. Therefore, stretching the soft RFID tag changes the electric length of the antenna; each infinitely small change in electric length shifts the resonant frequency. Now, if we send signals to RFID tags, the frequency of the carrier signal can be changed to the specific resonant frequency of the antenna's current electric length. The backscattered signals from the RFID tags will indicate phase and amplitude changes due to the resonant frequency.

#### Antenna matching

As mentioned in the previous section, the RFID antenna utilizes power from the incoming electromagnetic waves to supply power to the RFID chip. *Impedance matching* between the RFID chip and the antenna is paramount in RFID antenna design because it affects how much power the antenna is able to reflect, hence determines the antenna's operational range.

**FIG. 3.**
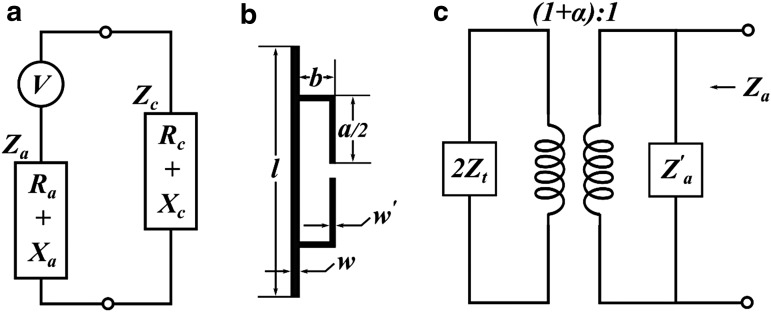
**(a)** Equivalent circuit of the RFID chip and the transporter antenna. *Z_a_* is the total impedance of the transport antenna, whereas *Z_c_* is impedance of the RFID chip. **(b)** Schematic diagram of T-matching for a dipole antenna. The *l* represents the length of the original dipole antenna. This antenna is connected at the center to a short second dipole. The close distance between matching stub and the original antenna is *b*, whereas *w* and *w'* are the widths of the original dipole antenna and the mating stub, respectively. **(c)** Equivalent circuit at the source point of the dipole antenna after T-matching. *Z'_a_* is the impedance of the first dipole antenna without T-matching, whereas *Z_t_* is the impedance that the matching stub created. The α in the ratio is the current division factor between the two connectors.

Figure 3 indicates how to match impedances. The best impedance matching for maximum power transmission is to conjugate impedance matching, which means ${R_a} = {R_c}$ while ${X_a} = - {X_c}$. The RFID chip we used (Monza R6, Impinj) possesses an impedance of ${Z_c} = \left( {13.5 - j \;126} \right) { \rm{ \Omega }} \;$ at 860 MHz. Therefore, we designed the transporter antenna to match the impedance of this RFID chip.

The theoretical impedance of a half-wave dipole antenna in free space is 73 + *j*42.5 Ohms,^48^ which makes it impossible to match the impedance of the RFID chip. Therefore, we used the T-match method^49^ to match the impedance between transporter antenna and RFID chip. The results shown in Figure 3b and c indicate that a half-wave dipole antenna can be changed through a centered short-circuit stub. The antenna *source* is connected to a small dipole of length $a \le l$ and is placed at a close distance *b* from the first and larger dipole. The electric current distributes along the two main radiators according to the size of their transverse sections. The impedance *Z_a_* at the source points is given by Equation 1:

\begin{align*}
 { Z_a } = { \frac { 2 { Z_t } { { \left( { 1 + \alpha } \right) } ^2 } Z_a^ \prime }  { 2 { Z_t } + { { \left( { 1 + \alpha } \right) } ^2 } Z_a^ \prime } } . \tag { 1 } 
\end{align*}

In Equation 1, *Z′_a_* is the original dipole impedance taken at its center in the absence of a T-match connection, whereas *Z_t_* is the impedance created by the matching stubs. $\alpha$ is the current division factor between the two connectors, $\alpha = \ln \left( {b / re^{\prime} } \right) / { \rm{ln}} \left( {b / re} \right)$. *re* and *re'* are the equivalent radii of the initial dipole and the matching stub.

We performed impedance calculation with finite element method (FEM) simulations with Computer Simulation Technology (CST) Microwave Studio. FEM includes the use of mesh generation techniques for dividing a complex structure into a large number of small elements. Calculations are made for every single element, and combining the individual results can give us the conclusive results of the structure. We modified the edge of the antenna to be asymmetric, so that created *l_m_* in Figure 1a, for a final fine matching step.

## Materials and Fabrication Methods

**FIG. 4.**
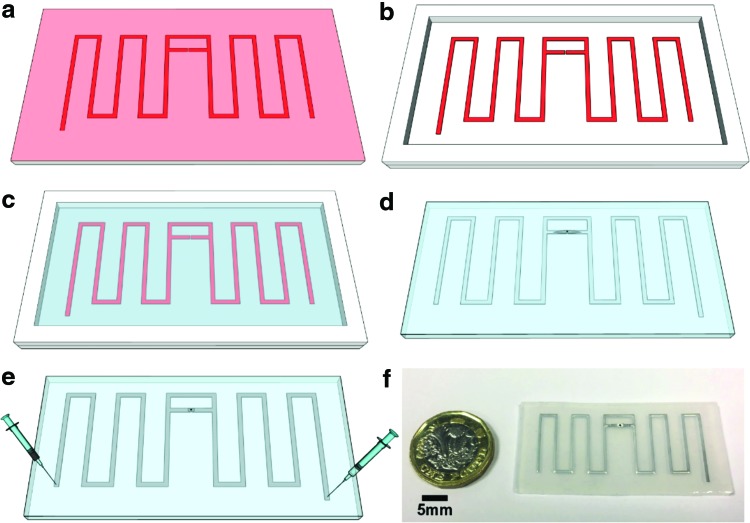
Fabrication process and prototype of the stretchable microfluidic sensor (RFID tag antenna). **(a)** Cut the sensor profile on a piece of self-adhesive vinyl attached on an acrylic substrate. Notice: the *red* and *pink* parts are a same piece of vinyl. Two different colors are used here to show the laser-cut profile clearer. **(b)** The unwanted part of vinyl was peel off, a 2 mm-thick acrylic frame was stuck on the substrate to form a soft-lithography mold. **(c)** Uncured Ecoflex with a controlled height of 2 mm was poured into the soft-lithography mould. **(d)** The casted piece of Ecoflex was sealed with another 0.15 mm Ecoflex film, and the RFID chip bonded on aluminum film was inserted into the channels. **(e)** Two needles were used to inject Galinstan into the channel. **(f)** Photograph of the final prototype.

Figure 4 shows the soft-lithography fabrication process of our stretchable antenna (photographs of the fabrication process are shown in Supplementary Figure S2). We designed and fabricated an antenna mould with a laser micromachining system (Protolaser U3; LPKF) and a self-adhesive vinyl film (CRAFTRKZO; d-c-fix^®^), as shown in Figure 4a. A piece of vinyl film was attached to an acrylic substrate (2 mm Acrylic Cast; AMARI). The channel profiles were cut with the LPKF laser system. We peeled the vinyl residuals (negative) from the substrate and laid a 2 mm-thick acrylic frame along the mould edges as indicated in Figure 4b.

We synthesized Ecoflex 00-50 (Reynolds Advanced Materials; 1:1 ratio), degassed it in a vacuum chamber, and poured the soft polymer into the mould as illustrated in Figure 4c. We put the mould into a convection oven at 65°C for 20 min and then removed the soft structure from the mould and flipped it over. A 150 μm-thin Ecoflex 00-50 layer was fabricated with spin coater to seal the unfolded channels, as shown in Figure 4d.

We made small incisions in the center of the thin Ecoflex layer to embed the RFID chip; the RFID chip (Monza R6; Impinj) was bonded on an Aluminum film with a bonding machine (FB-300 manual RFID wrapping machine) and inserted in the channels. During the bonding process, the chip was aligned to the substrate with a vacuum sucker, microscopes, and cameras in the bonding machine, an adhesive material was dispensed between the chip and the aluminum film, then the chip was firmly bonded on the aluminum film with hot-pressing for 10 s.

We inserted two injection needles into the soft structure, as illustrated in Figure 4e. One needle was used to inject the Galinstan into the channel, and the other needle was used to release air pressure. The Galinstan made contact with the aluminum pads of the RFID chip without leaking to the surface. At the end, we applied a thin film of uncured Ecoflex on top of the device to seal the open holes created by chip insert and liquid metal injection.

## Experimental Design

**FIG. 5.**
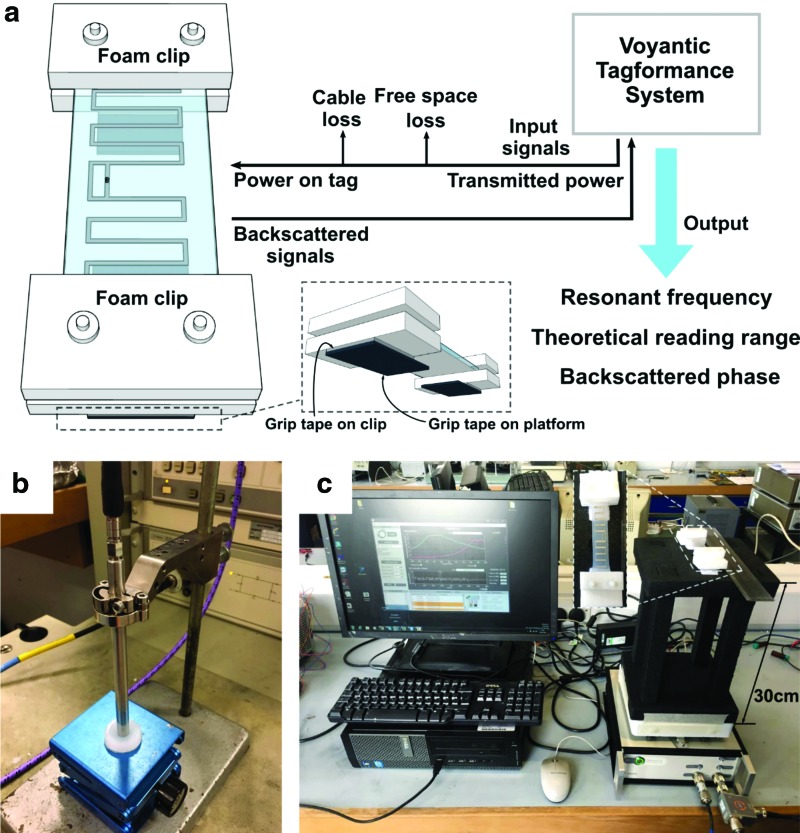
**(a)** We used the Voyantic Tagformance system to measure characteristics of the proposed antenna. This measurement system sends input signals to the tag antenna with transmitted power. The power received on tag is the transmitted power minus cable loss and free space loss. RFID chip in the tag antenna receives the input signals, absorbs energy in the signals, and then sends backscattered signals back to the measurement system. According to the results of the measurement system, we can know the resonant frequency and the theoretical reading range of the tag antenna, as well as the phase of the backscattered signals. We affixed the two edges of the tag antenna with clips made of rigid foam. To stretch the tag antenna and hold its position for measurement, we stuck grip tape on the surface of the foam clips and on the measurement platform. **(b)** Experimental setup for measurement of the permittivity of Ecoflex 00-50 with the Agilent 85070E dielectric probe kit system. **(c)** Photograph of the experimental setup of the soft antenna and the Voyantic Tagformance system.

The permittivity of Ecoflex 00-50 and the resistivity of Galinstan are parameters that are required to simulate *S_11_*; *S_11_* is the input reflection coefficient or input return loss. *S_11_* indicates the radiation efficiency of the antenna and reaches its maximum value at resonant frequency. We used an Agilent 85070E dielectric probe kit to measure the permittivity variations of Ecoflex 00-50 in a frequency range from 200 MHz to 1.2 GHz, as shown in Figure 5b. We simulated changes in resonant frequency and radiation efficiency under strain variations between 0% and 50% and a step size of 5%.

We used a Voyantic Tagformance system to measure the characteristics of our antenna design; namely the frequency modulation of our soft RFID sensor due to strain variations, as indicated in Figure 5a and c. This setup allowed us to determine the resonant frequency, the theoretical RFID reading range, and the phase changes of the backscattered signals for every applied strain in the range from 0% to 50% with a step size of 5%.

We repeated each measurement seven times to indicate the soft polymer's reversibility. We fabricated three sensor samples to enable us to evaluate the repeatability of our fabrication process. We did a cyclic stretching test to prove the reliability of the proposed sensor when it is stretched to 50% for 240 times. It is worth mentioning that we calculated the strain value in all the experiments with $\left( { \left( { { \frac { \Delta { l_x } }  { { l_x } } } } \right) \times 100 { \rm { \% } } } \right)$, *l_x_* and Δ*l_x_* are shown in Figure 1. The length we used in calculation is the effective antenna length in the *x*-direction, not the distance between the foam clips. We also tested the soft RFID sensors when we applied twists of 0°, 45°, and 90°.

**FIG. 6.**
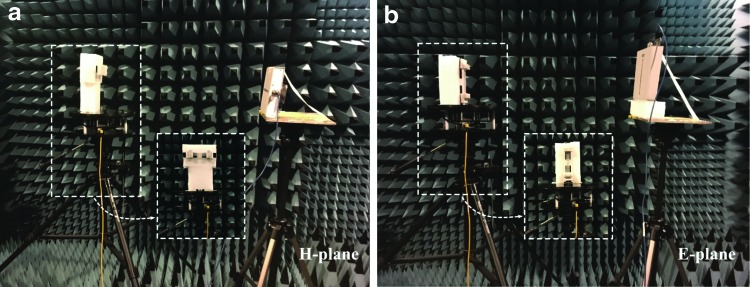
Radiation pattern measurement in the **(a)** H-plane and the **(b)** E-plane in an anechoic room with Diamond Engineering Automated measurement system and an automatic turntable. In the measurement, the reader antenna holds its position while the strain sensor (transporter antenna) turns with the turntable.

We used a diamond engineering automated measurement system and an automatic turning table in an anechoic room to measure the radiation patterns of our soft RFID sensor in the H- and E-planes (Fig. 6). An anechoic room absorbs reflections from acoustic and electromagnetic waves. We analyzed the directivity of the antenna (in the H- and E-planes) when subjected to strains of 0%, 25%, and 50%.

## Results

### Permittivity of Ecoflex 00-50

**FIG. 7.**
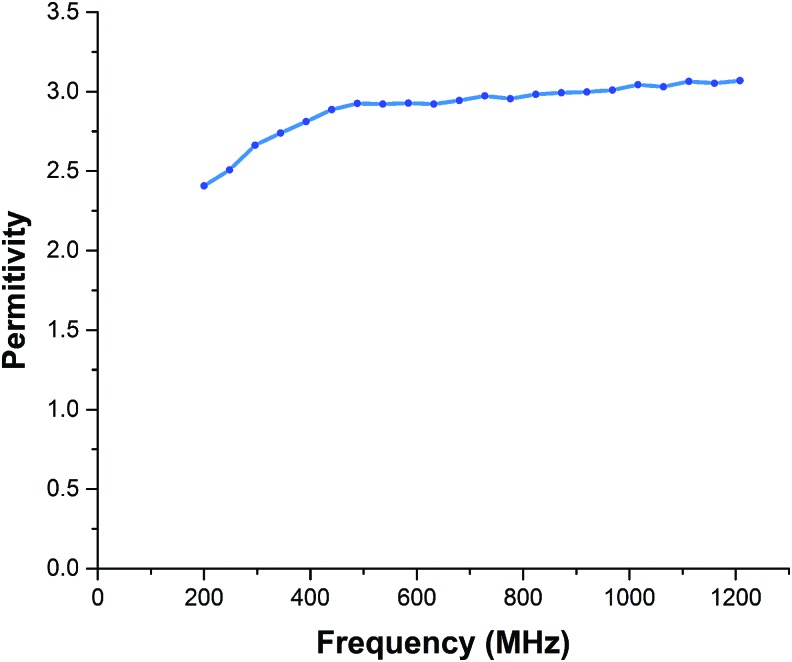
Permittivity of Ecoflex type 00-50 in the frequency range of 200 MHz–1.2 GHz. Measured with Agilent 85070E dielectric probe kit system.

According to the results in Figure 7, the relative permittivity (${ \varepsilon _r}$) of Ecoflex 00-50 is nearly stable at 3 in the range of 500 MHz–1.2 GHz, as shown in Figure 7. The operating frequency range of our sensor is 800–860 MHz, which is covered in this stable range. Thus, we can take ${ \rm{ \;}}{ \varepsilon _r} \;$ of Ecoflex as a constant in calculation and simulation.

### Simulation of antenna efficiency

When our soft RFID sensor is stretched, its antenna parameters such as resonant frequency and radiation efficiency change. Below, we analyze, analytically, the antenna parameters due to strain variations and we apply an ideal folded half-wave dipole antenna model.

The resonant frequency of a half-wave dipole antenna^50^ is given by

\begin{align*}
 { f_r } = \frac { c }  { { 2 { l_e } \sqrt { { \varepsilon _e } } } } \times r , \tag { 2 } 
\end{align*}

where *l_e_* is the effective electrical length of the antenna; *f_r_* is the resonant frequency; ${ \varepsilon _e}$ is the effective permittivity of the antenna substrate; and *r* is the correction factor of a folded half-wave dipole antenna. We can estimate the effective relative permittivity through the study of Jackson *et al*.^51^:

\begin{align*}
 { \varepsilon _e } = 1 + \left( { \frac { { { \varepsilon _r } - 1 } }  { 2 } } \right) { \frac { { K_1 } }  { { K_2 } } } , \tag { 3 } 
\end{align*}

where ${ \varepsilon _r}$ is the relative permittivity of Ecoflex, which is considered a constant here; ${ K_1 } = { \frac { K \left( { { k_1 } } \right) }  { K^{\prime} \left( { { k_1 } } \right) } }$ and ${ K_2 } = { \frac { K \left( { { k_2 } } \right) }  { K^{\prime} \left( { { k_2 } } \right) } }$ while $K \left( k \right)$ is a complete elliptic function of the first kind and $K \left( {k^{\prime} } \right)$ is its complimentary function.

In the K function, ${ k_1 } = { \frac { { l_e } }  { { l_e } + 2s } }$ and ${ k_2 } = { e^ { - { \frac { \pi s }  { 2h } } } }$, whereas *l_e_* is effective electrical length of the antenna, *s* is the gap at the feed point, and *h* is height of substrate of the antenna. According to Equations 2 and 3, resonant frequency of a half-wave dipole antenna will change when its effective electrical length or its substrate height changes.

Equation 2 shows the resonant frequency of a half-wave dipole antenna changes with its effective electrical length, whereas Equation 3 indicates that the resonant frequency also changes with the substrate height of the antenna.

Rigid antennas possess a fixed effective electrical length, whereas our soft antenna's effective electrical length changes due to strain. Due to the Poisson effect, our antenna physically changes according to

\begin{align*}
 { \frac { \Delta { l_y } }  { { l_y } } } = 1 - { \left( { 1 + { \frac { \Delta { l_x } }  { { l_x } } } } \right) ^ { - \nu } } , \tag { 4 } 
\end{align*}

where *l_x_* is the antenna length; $\Delta {l_x}$ describes a change in antenna length; *l_y_* is the antenna width; $\Delta {l_y}$ describes a change in antenna width; and the initial (not stretched) electrical length of our antenna is ${l_x} + 10{l_y} + {l_m}$. Since${ \rm{ \;}}{l_m} \ll {l_x} + 10{l_y}$, we neglect the effect of ${l_m}{ \rm{ \;}}$ here. Figure 1 complements Equation 4.

We insert Equations 3 and 4 in Equation 2 and receive a new formulation for the resonant frequency for our soft antenna

\begin{align*}
 { f_r } = \frac { c }  { { 2 \left( { { l_x } \left( { 1 + { \frac { \Delta { l_x } }  { { l_x } } } } \right) + 10 { l_y } \left( { 2 - { { \left( { 1 + { \frac { \Delta { l_x } }  { { l_x } } } } \right) } ^ { - \nu } } } \right) } \right) \times \sqrt { 1 + \left( { \frac { { { \varepsilon _r } - 1 } }  { 2 } } \right) { \frac { { K_1 } }  { { K_2 } } } } } } \times k , \tag { 5 } 
\end{align*}

where $\nu$ is the Poisson ratio of Ecoflex type 00-50.

**FIG. 8.**
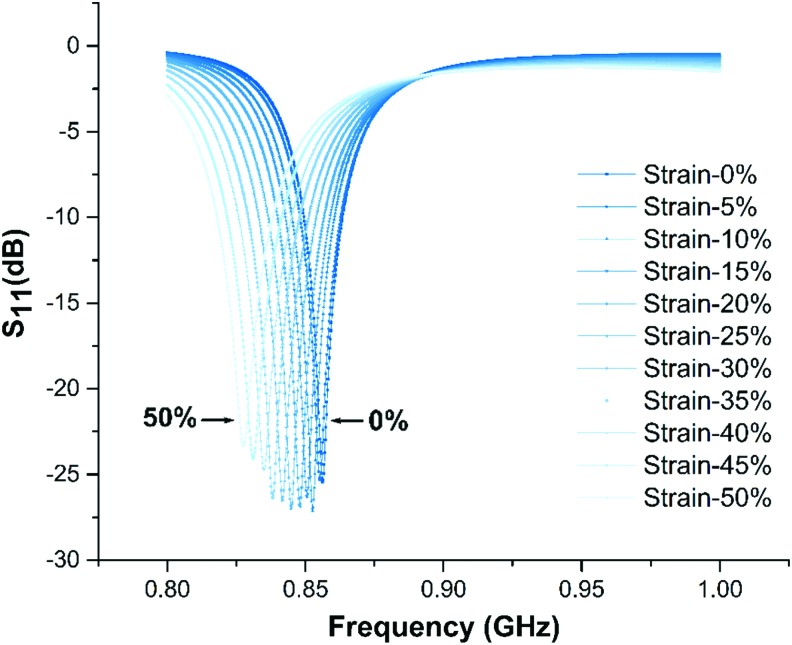
Finite elements simulation results. Under 0% strain condition, simulated resonant frequency of the antenna is 856 MHz. The simulated resonant frequency continuously goes down to 827 MHz when the antenna is under 50% strain.

We performed an electromagnetic finite element simulation using CST to determine *S_11_*, the input return loss, under a variety of strains. *S_11_'*s maximum indicates the antenna's resonant frequency. Figure 8 indicates the simulation results; the soft antenna has been strained from 0% to 50% with a step size of 5%; the *S_11_* peak values are all below −20 dB at resonance frequency for each strain condition indicating that 99% of the power from the input signal is scattered back from the soft RFID sensor. We successfully matched antenna to RFID chip resistance.

### Resonant frequency upon strain

The Voyantic Tagformance measurement system controls the power of the signal that we transmit to the soft RFID sensor. First, we set the initial signal power to 25 dB. Second, we gradually decreased the signal power until we could not detect the backscattered signal from the soft RFID sensor anymore. Power of the input signal was recorded as Transmitted Power (*P_t_*). We repeated this process for each sampling frequency in the frequency band of 800–880 MHz, with a step of 1 MHz.

As indicated in Figure 5a, the power received by the soft RFID tag antenna (${P_{tag}}$) can be calculated as

\begin{align*}
{P_{tag}} = {P_t} - {L_c} + {G_t} - FSPL , \tag{6}
\end{align*}

where *L_c_* are cable losses; *G_t_* is the Gain of the Voyantic Tagformance's transmitter antenna; and *FSPL* are losses due to free space transmissions. ${L_c} , {G_t} , FSPL$ can be determined by calibrating the Voyantic Tagformance.

**FIG. 9.**
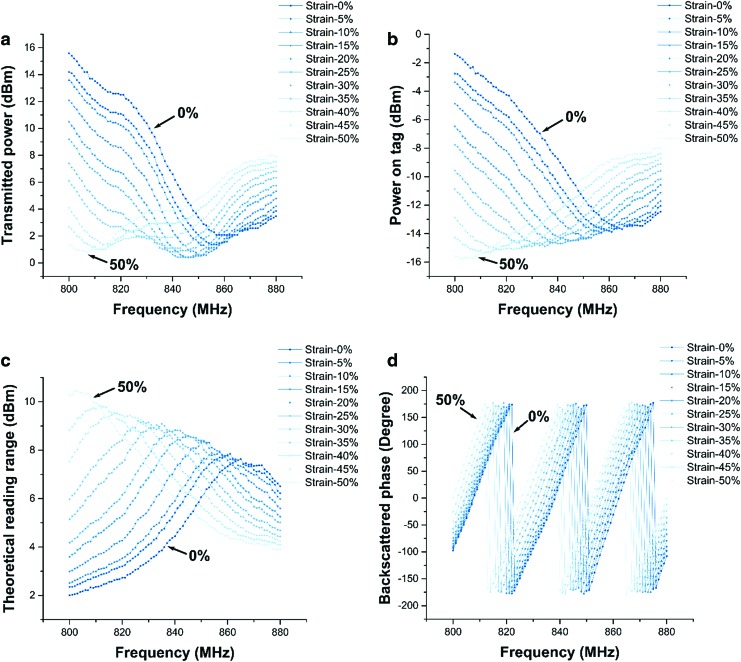
**(a)** Transmitted power. The minimum transmitted power recorded for the sensor under 0%–50% strain, with a step of 5%. **(b)** The power received on tag, which was calculated based on the transmitted power and cable and free space loss. **(c)** Theoretical reading range forward, which was calculated based on the power received on tag. **(d)** Backscattered phase in the frequency band of 800–880 MHz (with a step of 1 MHz) when the sensor was under 0%–50% strain, with a step of 5%.

Figure 9a shows the minimum transmitted power (*P_t_*) for each sample frequency to result in a detectable backscattered signal from the soft RFID sensor. Figure 9b indicates the power received on tag antenna (power on tag, ${P_{tag}}$) corresponding to the minimum *P_t_* for each sample frequency.

The theoretical measurement reading range of the soft RFID sensor is frequency dependent and can be calculated by

\begin{align*}
 { R_r } = \sqrt { { \frac { { P_ { max , EIRP } } }  { { P_ { tag } } } } } \times \frac { c }  { { 4 \pi f } } , \tag { 7 } 
\end{align*}

where ${P_{max , EIRP}}$ is the maximum signal power of the Voyantic Tagformance (default: 3.28 W). ${P_{tag}}$ in this formula is related to the minimum transmitted power. Figure 9c depicts the reading ranges calculated with Equation 7 for the various frequencies; our soft RFID sensor has a reading range of ∼7.5 m, whereas this range increases if the soft sensor is under strain. The reading range of the sensor increases to 10 m under a strain of 50%. The resonant frequency decreases from 862 MHz in idle state (no strain) to around 802 MHz under 50% strain. Figure 9d illustrates the backscattered signals from the soft RFID sensor under different strain conditions.

### Repeatability and reliability test

**FIG. 10.**
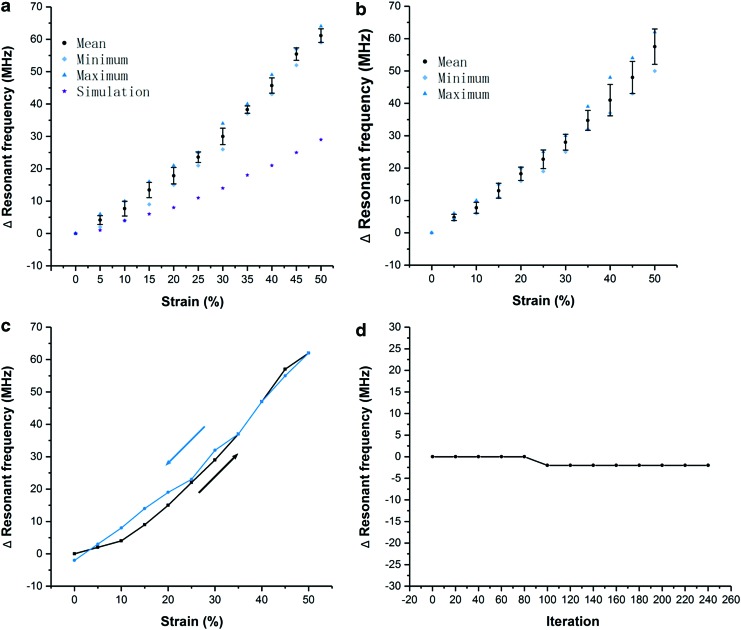
**(a)** Resonant frequency change upon different strain conditions. In the experiment, we did seven tests on the same prototype. Error bars represent standard deviation. **(b)** Resonant frequency change upon different strain conditions. In the experiment, we did one test on three different prototypes with the same design and same fabrication method. Error bars represent standard deviation. **(c)** Hysteresis test. A prototype was tested from 0% to 50% of strain, leave for 24 h, then from 50% back to 0%. **(d)** Cyclic stretching test. The sample was stretched to 50% of strain and then back to 0% for 240 times. Resonant frequency of the sample was measured each 20 times of stretching.

We tested the reliability of our soft RFID sensor by performing seven strain experiments. We measured the resonant frequency under 0%–50% strain, with a step size of 5%. Figure 10a shows the average value and standard deviation of the resonant frequency for each strain. The resonant frequency dropped ∼61 MHz (from 861 to 800 MHz) from 0% to 50% strain, whereas the maximum standard deviation did not exceed 2.6 MHz.

We performed a similar experiment in Figure 10c; once we reached 50% strain, we left the device stretched for 24 h in the laboratory before we gradually released the device to 0% strain. We also involved the simulation results of resonant frequency upon strain in Figure 10a as a comparison with the measurement results. In the simulation, the resonant frequency changed 29 MHz in total (from 856 to 827 MHz) from 0% to 50% strain, which is smaller than the measurement results.

We fabricated another three prototypes of the same sensor design and again performed strain-resonant-frequency tests. Average value and standard deviation of the resonant frequency for each prototype are shown in Figure 10b. Figure 10b shows similar results than Figure 10a. Figure 10d depicts a cyclic s stretching test to prove the reliability of the proposed sensor when it is stretched 240 times.

As shown in the results, the resonant frequency of the sensor remains stable in the test (only dropped 2 MHz between 80 and 100 of iteration). We also measured resonant frequency response of a soft RFID sensor due to twist rather than strain and put the results in Supplementary Data. As shown in Supplementary Figures S3 and S4, three different twist angles were tested. According to the results, the electrical characteristics of our soft RFID sensor at frequency of operation are highly sensitive to mechanical strain while having no response to twisting.

### Radiation pattern

**FIG. 11.**
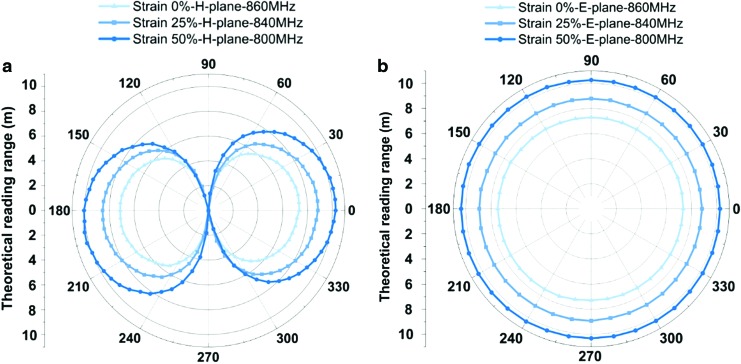
Theoretical reading range of the antenna in the H-plane **(a)** and the E-plane **(b)** with 0%, 25%, and 50% length stretching in the *x*-direction. The data were recorded for every 5° rotation in the H-plane and every 10° rotation in the E-plane.

We measured the theoretical reading range from our soft RFID sensor in the H- and E-planes with an antenna measurement system consisting of antenna measurement studio 5.5 and Diamond Engineering. The left subfigure of Figure 11 shows that omnidirectional radiation patterns were measured in the H-plane. The right subfigure of Figure 11 indicates that the antenna is constrained in directions that are parallel to the sensor's *x*-axis ($60^ \circ + n \pi < \theta < 120^ \circ + n \pi$; *n* is an arbitrary integer). This constraint might have to be considered in certain applications. The different strain conditions have an impact on our soft RFID sensor's reading range *radius*; if strain is applied, the reading range radius increases.

## Discussion

The results show that our soft RFID sensor changes its resonant frequency and the phase of its backscattered signal as function of strain.

Our measurements correlate with our simulations, the initial resonant frequency of all our sensor prototypes lies in the region of 860 MHz. In our simulation, the resonant frequency did not decrease as much under strain as in our experiments. This mismatch could be caused by the simplified model that we used for the Poisson effect or a potentially emerging resistance between the Galinstan channels and the RFID chip that might occur under strain.

We matched the resistances of the RFID chip and the antenna under 0% strain. Once we apply strain, the resistance of the antenna changes, hence the resistances of the RFID chip and antenna do not perfectly match anymore. Therefore, we expected a decrease in the measurement reading range with an increase in strain. However, the measurement reading range increased with an increase of strain.

One explanation could be that a folded dipole antenna consists of conductors with opposite current flows; the opposing magnetic fields that are evoked through the currents might cancel each other out. Now, if we stretch the soft antenna, the conductors are being further separated and the magnetic fields might be too far away to interfere with one another. This may enhance the radiation efficiency of the antenna. An increase of the radiation efficiency might outperform an imperfectly matched antenna-RFID chip.

In this study, we investigated the resonant frequency response of stretchable soft RFID sensors due to strain. Future work might focus on the design of soft RFID sensors based on phase shift differences that occur in backscattered signals. We also want to increase the initial resonant frequency of our soft RFID sensors to increase the strain range. RFIDs usually operate in the range of 800 MHz–1 GHz, whereas, currently, our system operates in the 800–860 MHz range.

## Conclusion

In this work, we designed and fabricated a stretchable strain sensor (soft RFID sensor) and tested a variety of its characteristics. The soft RFID sensor is a deformable folded half-wave dipole antenna. Its resonant frequency and backscattered signal phase changes under strain. We showed that our soft sensor is stable, reliable, and manufacturable. Our sensor consists of Ecoflex 00-50 and Galinstan, materials that are stretchable and withstand permanent deformation. The soft RFID sensor can be easily applied to for applications including wearable strain sensors or as part of soft robotic systems, as demonstrated in this study. The sensor does not require a power supply, indicates long measurement reading ranges (>7.5 m), and shows omnidirectivity.

## Supplementary Material

Supplemental data
