# Supplementary material for: Soft Radio-Frequency Identification Sensors: Wireless Long-Range Strain Sensors Using Radio-Frequency Identification
Source: Soft Robot. 2019 Feb 11;6(1):82–94. doi: 10.1089/soro.2018.0026 (PMC6386780; doi:10.1089/soro.2018.0026)
Supplement: Supplemental data [file Supp_Data.pdf]

# Supplemental Information

**Figure. S1**

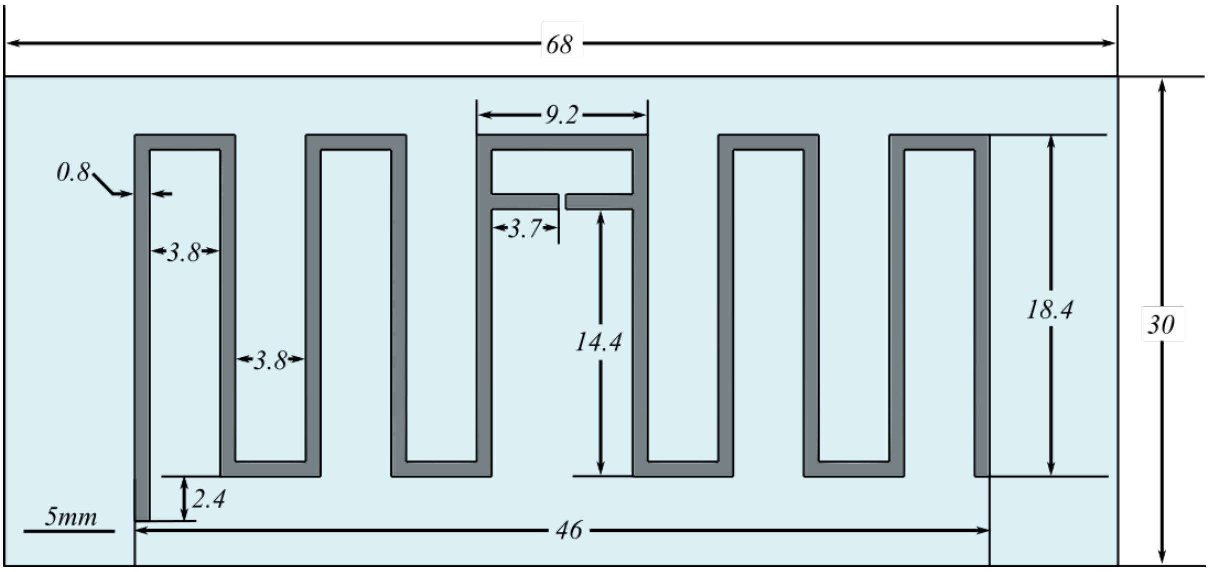


Figure S1. Dimensional drawing of the meander line antenna. The unit in this figure in milimetre. The height of the substrate is 2.15mm in total.

**Figure. S2**

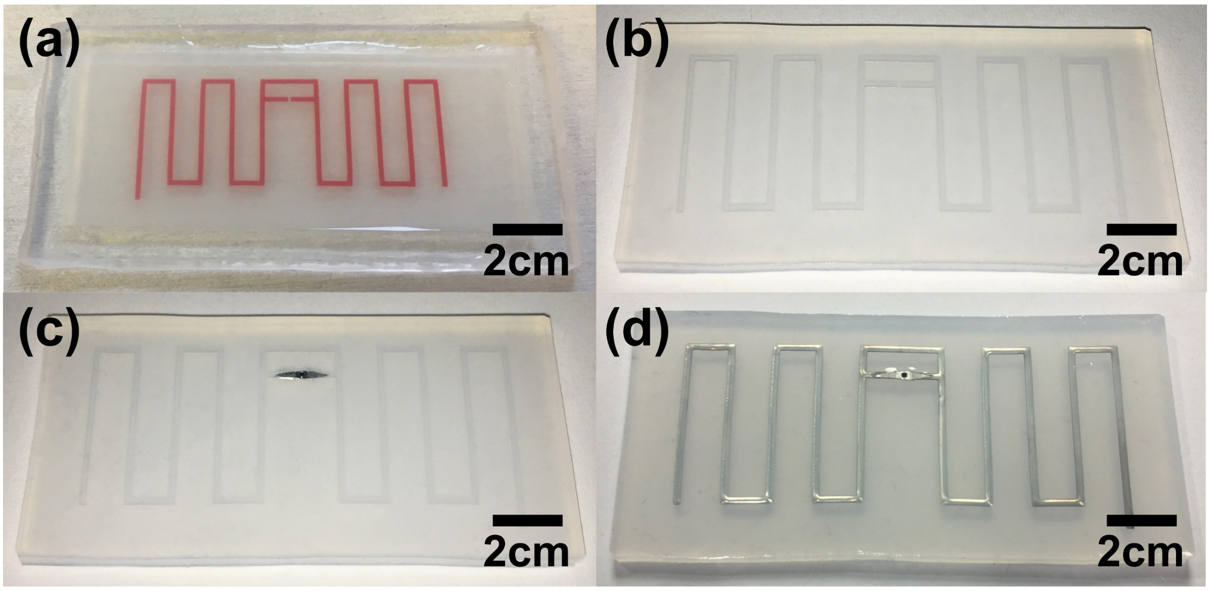


Figure S2. Photographs of the fabrication process.

**Figure. S3**

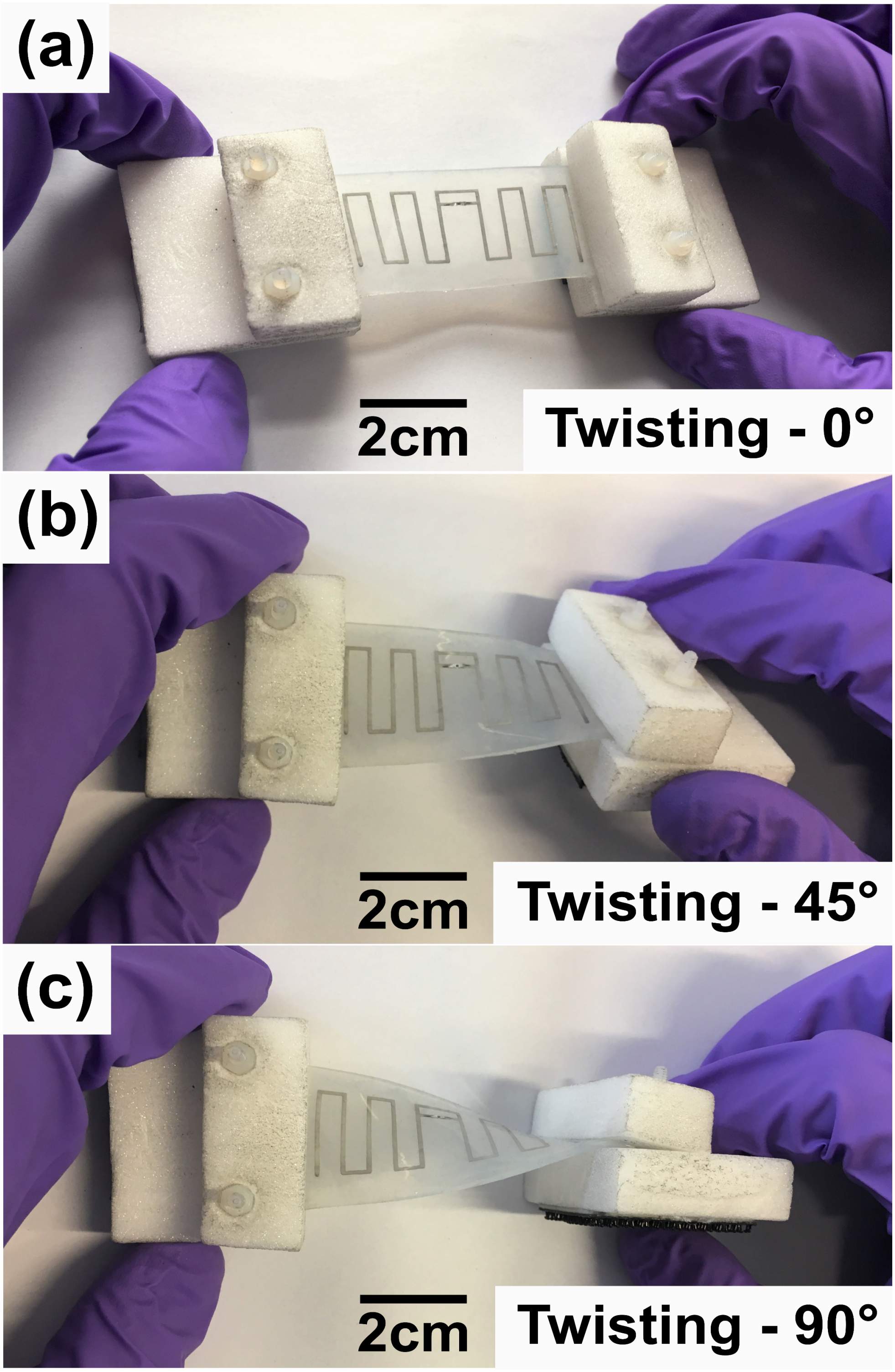


Figure S3. Demonstration of three different test conditions in the twisting experiment.

**Figure. S4**

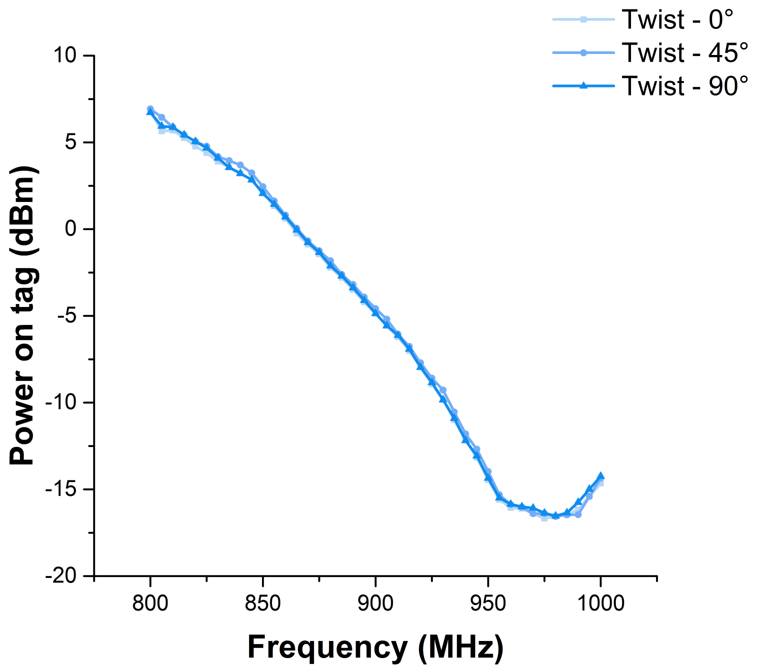


Figure S4. A prototype was tested under three different twist angles: 0° , 45° , and 90° .
